# Addressing asthma and obesity in children with community health workers: proof-of-concept intervention development

**DOI:** 10.1186/s12887-016-0745-0

**Published:** 2016-12-01

**Authors:** Molly A Martin, Steven K. Rothschild, Elizabeth Lynch, Katherine Kaufer Christoffel, Militza M. Pagán, Jose Luis Rodriguez, Anna Barnes, Kelly Karavolos, Antonieta Diaz, Lucretia M. Hoffman, Diana Plata, Sandra Villalpando

**Affiliations:** 1 University of Illinois at Chicago, 840 South Wood Street, M/C 856, Chicago, IL 60612 USA; 2 Rush University Medical Center, 1700 W Van Buren, Suite 470, Chicago, IL 60612 USA; 3 Lurie Children’s Hospital, 225 E Chicago Ave, Chicago, IL 60611 USA; 4 Puerto Rican Cultural Center, 2700 W Haddon Ave, Chicago, IL 60622 USA; 5 Greater Humboldt Park Community of Wellness, 1116 N. Kedzie, Chicago, IL 60651 USA; 6 University of Illinois at Chicago, Jane Addams College of Social Work, 1040 West Harrison Street, Chicago, IL 60607 USA; 7 Forsyth County Department of Public Health, 201 N Chestnut St, Winston-Salem, NC USA; 8 Rush University Medical Center, College of Medicine, 600 S Paulina St, Chicago, IL 60612 USA; 9 University of Illinois at Chicago, College of Medicine, 808 S Wood St, Chicago, IL 60612 USA

**Keywords:** Asthma, Obesity, Pediatrics, Community health worker

## Abstract

**Background:**

The objective of this study was to design and test the feasibility and impact of a community health worker (CHW) intervention for comorbid asthma and obesity.

**Methods:**

Using a proof of concept study design, we collected pre/post outcomes from a single intervention cohort of urban low-income in a single community area. A community-based participatory research approach was employed. Forty-six children and their caregivers were recruited. Children were 5–12 years old with physician-diagnosed asthma and body mass index (BMI) > 85%. Families were offered 12 home visits from CHWs that integrated asthma and obesity core curriculums. The primary asthma outcome was asthma control, measured via the Childhood Asthma Control Test (cACT). The primary obesity outcome was child body mass index (BMI).

**Results:**

Families received a median of 10 out of the 12 home visits over 1 year. At 1 year, there was a significant improvement in the number of children with controlled asthma as measured via cACT (85.7% at 1 year compared to 61.9% at baseline, *p* = 0.01). Activity limitations and emergency utilization were reduced while inhaler technique improved (*p* < 0.01 for all). Child BMI z-score was reduced: mean = 1.97 (SD 0.79) at 1 year compared to mean = 2.13 (SD 0.40) at baseline, *p* < 0.01. No association was seen between change in child BMI and change in asthma control. Worse baseline child depression scores were associated with less improvement in asthma control (*p* = 0.003) and higher baseline caregiver post-traumatic stress disorder scores were associated with increased child BMI (*p* = 0.012).

**Conclusions:**

The CHW intervention has promise for improving asthma and weight outcomes in high-risk children with comorbid asthma and obesity; this model warrants further development and investigation.

## Background

Obesity constitutes an important risk factor for asthma. Asthma prevalence is higher in overweight and obese children [1, 2] and obesity frequently precedes asthma development, suggesting a causal role [3, 4]. Obese children with asthma use more medicine, wheeze more, and have more unscheduled emergency department visits than non-obese children with asthma [4].

Despite these strong associations, interventions targeting children with both asthma and obesity are limited [5–9]. This gap in the field prompted the formation of the Community United to Raise Awareness: Asthma and Active Living study (CURA 2) with the goal of developing a behavioral intervention for children with both asthma and obesity. The home-based intervention used a community health worker (CHW) asthma model that had previously been tested in the target community [10]. The intervention was strengthened by increasing the dose and duration, and by adding pediatric obesity modules. The CURA 2 intervention was then tested in a proof of concept study to assess if these changes resulted in a feasible intervention that could improve asthma control and reduced body mass index (BMI).

## Methods

### Theoretical model

This study is part of the Rush Center for Urban Health Equity which adapts the Warnecke model for population health disparities [11]. Proximal, intermediate, and distal factors and their contributions to disparities are considered. The portion of the CURA 2 study described in this manuscript focused on the home and aimed to address family social relationships, risk behaviors, psychosocial factors, and the physical environment. Other study phases not described here targeted schools. Social Cognitive Theory guides the CHW intervention [12]. Behavior is shaped and maintained by consequences, particularly by immediate feedback from both objective sources (such as observation of inhaler technique or home triggers) and an individual’s social network (beliefs and traditions of family and friends). The asthma CHW home intervention measures and supports these behaviors and social networks using self-management skills (self-monitoring, social support, environmental rearrangement, problem solving, and behavior change plans).

### Study design

This proof of concept study was uncontrolled with data collection at baseline (pre) and 12-months (post) in the homes by research assistants (RAs), and 12 CHW home visits offered over the 12-month intervention period. A community-based participatory research approach was employed to design and oversee the study. The study built off of partnerships developed in previous research in a low income community area in Chicago with high documented asthma prevalence and morbidity [10, 13]. The study team included clinician researchers with expertise in behavioral interventions, asthma, obesity, and child development. Community partners on the study team included a local health coalition (Greater Humboldt Park Community of Wellness), a social service organization (Puerto Rican Cultural Center), a parent-led service organization (Women Living with Hope), and an obesity advocacy organization (Consortium to Lower Obesity in Chicago Children).

The recruitment goal for this uncontrolled cohort was 30–50 children that met the following inclusion criteria: 5–12 years old, residence within defined geographic boundaries, physician-diagnosed asthma (per caregiver report), overweight or obese (BMI > 85%), and not attending a school with a planned subsequent school-level intervention. Participants were recruited via community events, schools, and local clinics. Written informed consent from caregivers and child assent were obtained in the home by research assistants in the family’s preferred language (English or Spanish). The study was approved by the Rush University Medical Center Institutional Review Board and Chicago Public Schools Research Review Board.

### Outcomes

Outcomes were assessed by bilingual RAs in the home at baseline and 12-months. Survey questions were asked verbally to the caregiver/child with the exception of the mental health instruments which were self-administered. Families received $50 both at the baseline and 12-month data collections for a total of $100 by the end of the study.

#### Asthma control

Asthma control was determined using the Childhood Asthma Control Test (cACT) [14]. A score of >19 is considered uncontrolled [14]. The Expert Panel Report 3 guidelines to assess control using questions on symptoms, medication usage, missed activities, and prednisone use were also used [15]. Families self-reported emergency department visits and hospitalizations.

#### Asthma medications

RAs asked to see all of the children’s asthma medications. For children with an inhaled corticosteroid (ICS), adherence was determined using a medication dose counter placed on the inhaler that documented the number of times the inhaler was actuated daily (Doser CT, MediTrack, Inc., South Easton, MA) or using the ICS canister’s integrated dose counter [10, 16]. Children were asked to demonstrate their medication technique [17].

#### Home triggers

Home asthma trigger data were obtained by caregiver report, a visual home assessment, and objective measurement [18]. Families were asked about behaviors related to allergens such as dust mites, pets, roaches and rodents, and exposures to irritants such as cigarette smoke. The visual home assessment included visual and olfactory examination of the child’s bedroom, main living area, kitchen, bathroom, and heating source. Saliva was taken from children and tested for cotinine with an ELISA using a high sensitivity quantitative immunoassay (Salimetrics, Inc, State College, PA), with calibrator range 0.8–200 ng/mL, sensitivity 0.05 ng/mL. Salivary cotinine levels ranging from 1–7 ng/ml were coded as environmental tobacco smoke (ETS) exposure [19]. A positive report of a trigger from any one of the three data sources resulted in a positive score for that trigger.

#### Allergen sensitivity

Aeroallergen sensitivity of children was assessed in the home at baseline using the ImmunoCAP® Rapid Inhalant Profile 1, an in vitro semi-quantitative assay for measurement of allergen specific IgE in whole blood (http://www.phadia.com/en/Products/Allergy-testing-products/ImmunoCAP-Rapid/).

#### Child and caregiver anthropometrics

Height was measured using a tape measure and straight edge. Weight was measured using a calibrated portable digital scale. BMI was calculated as kilograms/meters squared using CDC age/sex specific growth charts formulas.

#### Child and caregiver hemoglobin A1c and lipid profiles

One caregiver per family and the child were asked to fast before the data collection visit, and blood was collected using dried blood kits. Hemoglobin A1c assays were performed using a standard procedure developed by ZRT laboratory [20]. Lipid profiles were measured using commercial kits (Randox Laboratory, UK) and reagents (e.g., enzymatic reagents, standards and control sera) [21].

#### Child physical activity

Children wore ActiGraph Accelerometers (GT1M and GT3X units) around their waists. Accelerometers were set for 15 s epochs and were worn for a minimum of 10 h/day for 4 days (including 1 weekend day). Physical activity levels were set using the Evenson cut points [22]. Children (or caregivers for children under 8) also completed a 7 day physical activity recall [23].

#### Home food environment

Visual inspection of the home food environment for obesogenic foods was conducted using the Home Food Inventory [24]. Caregivers were asked nutrition behavior questions from the Family Nutrition and Physical Activity screening tool [25]. Food security was assessed using the USDA Food Security Survey [26].

#### Other

Sleep disordered breathing was assessed using the snoring portion of the Pediatric Sleep Questionnaire [27]. Caregivers were asked about self-efficacy for asthma management using the Parent Asthma Self-Efficacy Scale [28]. A similar 10-item scale about self-efficacy nutrition and physical activity was created. Caregivers were screened for depression symptoms using the PHQ-9 [29] and for trauma using the Short Form of the PTSD Checklist - Civilian Version [30]. Children were screened for depression using the Child Depression Inventory 2 Short Form [31].

### Intervention

CHWs were trained by investigators using a 40 h curriculum that contained established asthma training [10] and newly created pediatric obesity modules (http://cuhe.rush.edu/Cura%202/Pages/CommunityHealthWorkerTraining.aspx.) Families were offered 12 home visits over 12 months from CHWs. Each home visit was expected to last about 60 min. Visits began with several minutes of social discussion for the purpose of connection. The CHWs approached each visit with the intention to teach one or two of the core curriculum topics (Table 1). The order of topics was developed based on the individual family’s interest and needs. The asthma and obesity curriculum topics were interconnected to address potential conflicts in messaging. For example, when discussing steroid medications, CHWs strongly emphasized that these are not the steroids associated with weight gain. When a CHW noted a barrier in the delivery or understanding of the education, she applied self-management skills. Behavior change plans were reviewed and new plans made at the end of each visit; behavior change plans are small goals for a specific change over a 2-week period. Examples include: “getting a prescription from my doctor” or “increasing exercise by 5 min a day”.

**Table 1:** Community health worker home visit topics

Asthma Core Curriculum	Obesity Core Curriculum	Self-Management Skills
General asthma facts	General obesity facts	Problem solving
Controller medications	Healthy foods	Social support
Inhalers and spacers	Beverages	Environmental rearrangement
Symptom recognition	Portions and labels	Self-monitoring
Asthma triggers	Physical activity	
Communicating with providers	Screen time	
Asthma and schools	Communicating with providers	
Asthma and obesity	Obesity and schools	

### Analysis

Basic summary statistics were calculated for baseline variables. Changes in the primary outcome of asthma control (cACT) from baseline to 1 year were assessed using McNemar’s exact test or paired samples *t*-test. Regression models were used to test the influence of dose, BMI, and baseline variables on primary asthma outcomes. Asthma secondary variables (Table 2) were tested for change from baseline to 1 year using McNemar’s exact test or t-test. Finally, variables with statistically significant changes were included in mediation analyses to determine their influence on primary asthma outcomes. These same series of analyses were repeated with the obesity primary outcome (child BMI z-score) and obesity secondary variables (Table 2).

**Table 2:** Clinical characteristics (*N* = 46)

Asthma control	
cACT, mean (SD)	20.9 (3.6)
cACT <20 (uncontrolled), N (%)	18 (40.0)
Uncontrolled in last 30 days, N (%)	
Day symptoms	17 (37.0)
Night symptoms	23 (50.0)
Excess quick reliever use	16 (34.8)
Activity limitations	16 (34.8)
Healthcare utilization for asthma over last 12 months, N (%)	
Emergency room	15 (32.6)
Hospitalization	6 (13.0)
Prednisone/prednisolone use	12 (26.1)
Medications	
Have a quick relief medication, N (%)	29 (63.0)
Have an inhaled corticosteroid, N (%)	20 (43.0)
Puffs/day inhaled corticosteroid, median (25th, 75th percentile)	0.6 (0.2, 1.4)
Have a spacer, N (%)	16 (34.8)
Percent correct technique, mean (SD)	58 (18.3)
Tobacco exposure, N (%)	
Self-reported smoker in the home	21 (45.7)
Cotinine level >1.0	11 (23.9)
Home triggers, N (%)	
Working vacuum	23 (50.0)
Dogs	17 (37.0)
Cats	4 (8.7)
Problem with roaches	18 (39.1)
Problem with rats/mice	14 (30.4)
Problem with mold	22 (47.8)
Allergen sensitivity, N (%)	
Dog	6 (18.0)
Cat	8 (24.0)
Dust mite 1 (*D. pteronyssinus*)	14 (42.0)
Dust mite 2 (*D. farina*)	13 (39.0)
Child anthropometrics	
Overweight (BMI 85–95%), N (%)	7 (15)
Obese (BMI ≥95%), N (%)	39 (84)
Child,^a^ N (%)	
Hemoglobin A1c borderline high (5.7–6.4)	16 (36)
Hemoglobin A1c elevated (≥6.5)	1 (2)
LDL cholesterol borderline high (110–129)	4 (9)
LDL cholesterol elevated (≥130)	6 (13)
HDL cholesterol borderline low (40–45)	8 (18)
HDL cholesterol low (<40)	18 (40)
Triglycerides borderline high^b^	6 (13)
Triglycerides elevated^b^	29 (64)
Child physical activity	
Minutes of moderate/vigorous physical activity, median (25th, 75th percentile)	39 (29,59)
Physical activity 60 min/day, N (%)	10 (21.7)
Self-reported physical activity,^c^ mean (SD)	2.8 (0.7)
Caregiver obese (BMI ≥30), N (%)	28 (60.9)
Caregiver,^d^ N (%)	
Hemoglobin A1c elevated (≥6.5)	10 (31)
LDL cholesterol elevated (≥160)	2 (5)
HDL cholesterol low (<40)	10 (23)
Triglycerides elevated (≥200)	17 (39)
Child screen time >2 hrs/day, N (%)	43 (93.5)
Child seems to stop breathing at night (obstructive sleep apnea), N (%)	10 (21.7)
Food security, N (%)	
Marginal	10 (23.0)
Low/very low	23 (50.0)
Percent of home food obesogenic, mean (SD)	35 (14)
Self-reported family nutrition in high risk category, N (%)	14 (30.4)

^a^ *N* = 45

^b^Age 0–9: 75–99 = borderline high, ≥100 = elevated. Age 10–19: 90–129 = borderline high, ≥130 = elevated

^c^A score of 1 indicates low physical activity, whereas a score of 5 indicates high physical activity

^d^ *N* = 44

## Results

The participating families (*N* = 46) were primarily low income and Hispanic which reflects the demographics of the target community (Table 3). Their clinical data are shown in Table 2. While only 40% of children had cACT scores indicating poor asthma control over the past 30 days, 74% were uncontrolled over the past year based on the EPR3 guidelines for control that include emergency room or hospitalization. Inhaled corticosteroid presence was low (43%) but only persistent asthma requires these medications. Quick relief medicines are essential for all children with asthma; however, 37% of children did not have a quick relief medication and 65% did not have a spacer. Technique with their devices was suboptimal. Across all devices, the mean number of demonstrated correct steps for usage was 58% (SD 18.3). Triggers in the home were common, as were allergies to common triggers. Almost a quarter of children had cotinine levels suggesting recent ETS exposure. The majority of children were obese (84%) and the rest overweight. Screen time exceeded recommendations (93.5% reported more than two hours per day) and few children (22%) met the requirements for 60 min/day of moderate/vigorous physical activity.

**Table 3:** Baseline demographics (*N* = 46)

Caregiver	
Female, N (%)	44 (95.7)
Age, mean (SD)	37.9 (7.6)
Years of school completed, median (Q1, Q3)	12 (9,13)
Hispanic, N^a^ (%)	39 (84.8)
Mexican	19 (48.7)
Puerto Rican	16 (41.0)
Other Hispanic	3 (7.7)
Refuse	1 (2.2)
Race, N^a^ (%)	
Black/non-Hispanic	7 (15.2)
White/non-Hispanic	3 (6.5)
Other	37 (80.4)
Place of birth, N (%)	
United States	18 (39.1)
Mexico	15 (32.6)
Puerto Rico	10 (21.7)
Other	3 (6.5)
If not born in US, years on mainland US, median (Q1, Q3)	15.5 (12, 24)
Income, N (%)	
< $5,000	9 (19.6)
$5,000–$14,999	12 (26.0)
$15,000–$19,999	14 (30.4)
$20,000–$59,999	11 (23.9)
Home ownership, N(%)	
Own outright/mortgage	5 (10.9)
Rent	36 (78.3)
Live with others rent free	4 (8.7)
Other	1 (2.2)
Marital Status, N(%)	
Married/live with partner	25 (54.3)
Single	18 (39.1)
Separate/Divorced/Widowed	3 (7.7)
Child	
Age, mean (SD)	9.7 (2.2)
Female, N (%)	31 (67.4)
Grade, median (Q1, Q3)	4 (2,5)
Place of birth, N (%)	
United States	42 (91.3)
Mexico	1 (2.2)
Puerto Rico	3 (6.5)
Child health insurance Medicaid, N (%)	44 (95.7)

^a^For race and ethnicity, participants were allowed to endorse multiple categories if they applied

Many psychosocial stressors were identified. Among caregivers, 28.9% had symptoms of moderate-to-severe depression, 66% had experienced at least one traumatic life event, and 33.8% screened positive for probable PTSD. In children, 5.3% had depression scores in the “high average” range while 13.2% in the “elevated” range.

**Fig. 1:**
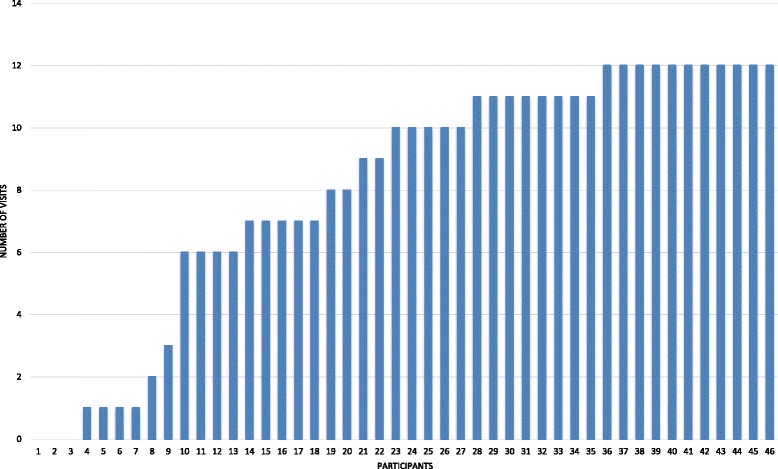
Completed CHW Visits in One Year

Families received a median of 10 out of the 12 home visits over 1 year (Fig. 1). Three families refused all home visits; one of these refused the 1 year data collection as well. Two other families accepted CHW visits but refused the 1 year data collection. As shown in Table 4, there was a significant improvement in children with controlled asthma as measured via cACT (85.7% at 1 year compared to 61.9% at baseline, *p* = 0.01) but this represented only a mean change in cACT score of 1.9 (SD 4.4). The minimally important difference for the cACT is 2 [32]. Activity limitations were reduced (7% at 1 year compared to 32.6% at baseline, *p* = 0.002) and emergency department/hospital use decreased (27.9% at 1 year compared to 53.5% at baseline, *p* = 0.003). Inhaler technique improved (90% correct at 1 year compared to 60% at baseline, *p* < 0.01). Child BMI z-score was reduced at 1 year: mean = 1.97 (SD 0.79) at 1 year compared to mean = 2.13 (SD 0.40) at baseline, *p* < 0.01. The number of CHW home visits received was weakly associated with healthcare utilization at 1 year (*p* = 0.049) but no other asthma outcomes were associated with number of visits received. Also, no association was seen between change in child BMI and change in asthma control (*p* = 0.188).

**Table 4:** Primary and secondary outcomes

	Baseline	One Year	*P* value
Asthma			
Asthma Control			
cACT^a^ <20	16 (38.1%)	6 (14.3%)	0.013
Uncontrolled day symptoms	16 (37.2%)	10 (23.3%)	0.109
Uncontrolled night symptoms	21 (48.8%)	11 (25.6%)	0.041
Excess quick reliever use	14 (32.6%)	13 (30.2%)	0.819
Activity limitations	14 (32.6%)	3 (7.0%)	0.002
ED/hospitalization in past year	23 (53.5%)	12 (27.9%)	0.003
Asthma self-efficacy, mean (SD)	4.1 (0.8)	4.4 (0.5)	0.006
Inhaler technique (% of steps correct), mean (SD)	60% (20)	90% (10)	<0.001
Obesity			
Weight control			
BMI z-score,^a^ mean (SD)	2.13 (0.40)	1.97 (0.70)	0.002
BMI percentile, mean (SD)	97.6 (2.2)	96.1 (4.4)	0.011
Obese (BMI ≥ 95%)	84%	74%	
Nutrition self-efficacy, mean (SD)	3.6 (0.8)	3.9 (0.7)	0.007
Child hemoglobin A1c, mean (SD)	5.6 (1)	5.3 (1.2)	0.027
Child HDL cholesterol, mean (SD)	44.9 (12.3)	52.3 (12.7)	0.013
Child sleep disordered breathing, mean (SD)	2 (2)	1.5 (1.8)	0.027
Caregiver BMI, mean (SD)	34.8 (9.6)	36.2 (10.1)	0.008
Caregiver hemoglobin A1c, mean (SD)	6 (1.3)	5.6 (1.8)	0.012
Caregiver HDL cholesterol, mean (SD)	50.2 (11.4)	54.7 (9.4)	0.036

^a^Primary outcomes

No significant changes from baseline to one year for presence of a quick relief or controller medication, controller medication adherence, prednisone use, cotinine level, observed triggers in the home, self-reported physical activity, screen time, accelerometry, food security, self-reported food in the home, observed obesogenic food in the home, child triglyceride, child LDL cholesterol, caregiver triglycerides, and caregiver LDL cholesterol

Secondary analyses were conducted to better understand the primary outcomes. None of the baseline asthma and obesity supporting variables predicted change in asthma control or child BMI. However, worse baseline child depression scores were associated with less improvement in asthma control (*p* = 0.003) and higher baseline caregiver PTSD scores were associated with more child BMI change in the direction of weight gain (*p* = 0.012). Many of the supporting variables did improve over the year, including nighttime asthma symptoms (*p* = 0.041), activity limitation (*p* = 0.002), ED/hospitalizations (*p* = 0.003), inhaler technique (*p* < 0.001), child hemoglobin A1c (*p* = 0.027), child HDL cholesterol (*p* = 0.013), and sleep disordered breathing (*p* = 0.027). Although caregivers were not the focus of the intervention, they gained weight (baseline BMI mean = 34.8, SD 9.6; 1 year BMI mean = 36.2, SD 10.1; *p* = 0.008) while showing improvements in hemoglobin A1c (*p* = 0.012) and HDL cholesterol (*p* = 0.036). However, none of the supporting variables that showed positive changes over the year mediated child asthma control or BMI. Caregiver depression decreased from baseline to 1 year (PHQ9 baseline mean = 7.5, SD 7.1; 1 year mean = 5.6, SD 6; *p* = 0.039). Change in caregiver depression score was weakly associated with change in child BMI z-score (*p* = 0.079) but not asthma control.

## Discussion

CURA 2 sought to test the feasibility of a home CHW intervention targeting both asthma and obesity, and to generate estimates for the potential of this intervention to improve child asthma control and BMI. The CHW intervention was well received. Only three families refused the CHW home visits. We strove to deliver 12 home visits but only 30% were able to complete all 12, and that was with difficulty. The primary content was intended to be delivered in visits one through ten, with visits 11–12 used for review and reinforcement. For asthma CHW intervention alone, Postma et al found that six visits were acceptable [33]. Campbell et al. showed that four home visits were associated with improved outcomes and a significant return on investment [34]. Because CURA 2 combined both asthma and obesity content, we suggest 10 home visits in a year would have been a more feasible goal.

While efficacy of the intervention cannot be definitively determined due to limited power and a lack of a control group, comparison of pre/post data suggest a possible benefit for asthma. The asthma control score, nighttime symptoms, activity limitations, ED/hospitalizations, and medication technique all improved at 1 year. However, the change in asthma control was modest and some of the children seemed to have mild intermittent controlled asthma from the start. Medication use and triggers in the home did not change, making it difficult to understand how changes in asthma occurred and whether they can be attributed to the intervention. Observed changes may simply be a result of natural improvements in asthma control as children grow. Future studies should consider restricting their sample to children with uncontrolled persistent asthma to reduce these limitations.

The outcomes for obesity were more robust. As children approach adolescence, it is typical for BMI to increase [35]. However, CURA 2 study participants had an overall decrease in BMI. A 0.25 BMI Z-score reduction is associated with reduced inflammation and improved insulin sensitivity in children; [36] the mean reduction in this study was 0.16. We saw some improvements at 1 year in markers of diabetes and lipids (for children and their caregivers) as well as obstructive sleep apnea, but we did not see anticipated changes in food environment, screen time, or activity level. The CHW intervention targeted healthy foods, screen time and physical activity as steps toward weight management which makes the mechanism for BMI reduction more difficult to understand and limits our ability to attribute it to the intervention.

Several key barriers to implementing the asthma control and obesity reduction strategies were identified. The first was lack of communication between the CHWs and clinical providers. CHWs (and potentially providers) could have benefitted from the ability to discuss care plans with providers. Second, mental health issues were prevalent; caregivers endorsed high levels of trauma and depression, and these variables appeared to have mediating effects on child BMI. Poorer psychological functioning has been associated with increased asthma morbidity [37] and similar associations are also seen in obesity [38, 39]. The impact of the CHW intervention on psychological functioning remains unclear [40] and requires further investigation.

## Conclusions

While the small sample size and lack of a control group limits our interpretation of intervention effects, we demonstrated feasibility and potential effects of a CHW intervention for children with both asthma and obesity. CHW asthma interventions have already shown benefits in asthma symptom control and healthcare utilization [41]. Our study is novel in that the intervention targets a subpopulation of children with both asthma and obesity. These children typically suffer worse asthma morbidity and their asthma is more challenging to control, [1, 2] yet few studies have published interventions specifically targeting these children [5–9]. None of these used CHWs and no pediatric obesity CHW training curriculum had been published to date. Therefore we created a curriculum, merged it with asthma training, and then delivered the combined asthma/obesity intervention to a high risk cohort of urban children and their families. For asthma medication adherence, medication technique, and triggers, outcomes were measured objectively; this limits the sometimes inaccurate results generated from self-reported data. The measured changes in asthma control and child BMI, while not large, warrant further study. Our small study could not address the full spectrum of social, behavioral, and environmental contributors to asthma and obesity [11, 41]. However our findings suggest that the CURA 2 CHW intervention, which could be expanded to include clinical and community partners and mental health resources, has promise for improving asthma and weight outcomes in the high-risk population of children with comorbid asthma and obesity.

## Abbreviations

CURA 2: Community united to raise awareness: asthma and active living
BMI: Body mass index
cACT: Childhood asthma control test
CHW: Community health worker
ETS: Environmental tobacco smoke
ICS: Inhaled corticosteroid
PTSD: Post-traumatic stress disorder
RAs: Research assistants
